# Advances in the rehabilitation of intensive care unit acquired weakness: A case report on the promising use of robotics and virtual reality coupled to physiotherapy

**DOI:** 10.1097/MD.0000000000020939

**Published:** 2020-07-10

**Authors:** Antonino Chillura, Alessia Bramanti, Francesco Tartamella, Maria Francesca Pisano, Elvira Clemente, Marzia Lo Scrudato, Giuseppe Cacciato, Simona Portaro, Rocco Salvatore Calabrò, Antonino Naro

**Affiliations:** a Spoke Center, PO V.Emanuele, Salemi (Trapani), IRCCS Centro Neurolesi; b IRCCS Centro Neurolesi Bonino Pulejo, Messina, Italy.

**Keywords:** critical illness polyneuropathy, intensive care unit acquired weakness, post-ICU rehabilitation, robotic neurorehabilitation, virtual reality rehabilitation

## Abstract

**Introduction:**

Traditional physiotherapy is currently the best approach to manage patients with intensive care unit acquired weakness (ICUAW). We report on a patient with ICUAW, who was provided with an intensive, in-patient regimen, that is, conventional plus robot-assisted physiotherapy. Aim of this case study was to assess the efficacy of a combined approach (conventional plus robot-assisted physiotherapy), on muscle strength, overall mobility, and disability burden in a patient with ICUAW in post-ICU intensive rehabilitation setting.

**Patient concerns:**

A 56-years-old male who was unable to stand and walk independently after hospitalization in an Intensive Care Unit. He initially was provided with daily sessions of conventional physiotherapy for 2 months, with mild results.

**Diagnosis:**

The patient was affected by ICUAW.

**Intervention:**

Given that the patient showed a relatively limited improvement after conventional physiotherapy, he was provided with daily sessions of robot-aided training for upper and lower limbs and virtual reality-aided rehabilitation for other 4 months, beyond conventional physiotherapy.

**Outcomes:**

At the discharge (6 months after the admission), the patient reached the standing station and was able to ambulate with double support.

**Conclusions:**

Our case suggests that patients with ICUAW should be intensively treated in in-patient regimen with robot-aided physiotherapy. Even though our approach deserves confirmation, the combined rehabilitation strategy may offer some advantage in maximizing functional recovery and containing disability.

## 1 Introduction

Intensive Care Unit acquired weakness (ICUAW) refers to “a wide variety of disorders characterized by acute onset of neuromuscular impairment for which there is no other plausible cause than the critical illness, greater than that resulting from prolonged bedridden, and typically associated with multiorgan failure.”^[1]^ Severe sepsis, acute respiratory distress syndrome, multisystem organ failure, prolonged and difficulty weaning from mechanical ventilation, hyperglycemia, corticosteroids, and neuromuscular blockers are known risk factors associated with ICUAW.^[1]^ Muscle weakness on awakening in ICU can be found in about 26% to 65% patients who underwent mechanical ventilation.^[2]^ In particular, it has been reported that the incidence of muscle weakness is proportional to the ventilation duration period.^[2]^

Clinical features of ICUAW include proximal and/or distal flaccid weakness with usually associated deep tendon hyopeflexia, muscle hypotrohy, distal sensory loss, and ventilator failure, depending on the clinical phenotype (critical illness polyneuropathy, myopathy, and neuromyopathy, that is, a combination of both).^[2]^ Facial nerve are usually spared.^[2]^ ICUAW entails also long-term consequences attributed to proximal weakness, loss of muscular mass, and fatigue, including physical, mental, and cognitive dysfunctions (as a part of post intensive care syndrome).^[3]^ Such functional impairment has been reported as severe in about 28% of patients,^[3]^ and persistent up to 5 years in acute respiratory distress syndrome survivors, with a prominent involvement of physical functioning, including physical status, activities of daily life, fatigue, and muscle weakness.^[4]^

Early rehabilitation approach in ICU mainly consists of mobilization, intensive insulin therapy, and electrical muscle stimulation. This has been reported as safe and feasible to improve patient's outcome (including functional status, muscle strength, quality of life or healthcare utilization outcomes), as well as to prevent deep vein thrombosis and venous stasis.^[5–8]^ Instead, there is few evidence to support post-ICU rehabilitation for post-critical illness patients,^[8]^ even though rehabilitation for adult survivors of critical illness has been considered a key strategy in improving functional recovery since many years.^[9]^

There is growing evidence on the feasibility and efficacy of rehabilitation robotics (using devices, exercise scenarios, and control strategies) aimed at facilitating the recovery of impaired sensory, motor, and cognitive skills.^[10,11]^ Conversely, there is currently no evidence on the usefulness of neurorobotics in managing ICUAW. Herein, we report the effects of an intensive neurorehabilitation program employing robot-aided physiotherapy to maximize the functional recovery and limit the permanent disabilities of a patient with ICUAW following acute respiratory distress syndrome.

## 2 Case report

A 56-years-old male suffering from hypertension and type 2 (non-insulin-dependent) diabetes mellitus (without relevant complications) was admitted to the Emergency Room of the S. Antonio Abate Hospital (Trapani, Italy) for severe acute respiratory failure, on early February 2018. He complained of fever and dyspnea for about 1 week, but his condition dramatically worsened the day before the Emergency Room admission. He was diagnosed with bilateral bronchopneumonia and transferred to ICU for intubation and mechanical ventilation. During ICU stay, he presented with a septic shock, refractory hypoxemia due to severe acute respiratory distress syndrome and even acute kidney failure, despite an adequate pharmacological therapy and mechanical ventilation. Veno-venous extracorporeal membrane oxygenation for refractory hypoxemia and hemodialysis were thus started. Specific laboratory tests revealed a positivity to H1N1 Flu Virus. His clinical condition slowly improved, so that he was weaned from veno-venous extracorporeal membrane oxygenation and hemodialysis within some days.

The patient was then admitted to our Neurorehabilitation Unit (28 February 2018). At admission, he had distal flaccid quadriplegia with deep tendon areflexia and stocking-glove anesthesia, without cranial nerve involvement. He spontaneously breathed through tracheostomy. He did not complain of significant pain. Muscle Research Council (MRC) (which grades muscle power on a scale of 0 to 5 in relation to the maximum expected for that muscle) was 5/60 (specifically, 3 muscles were tested in each extremity using the 0–5 MRC scale).^[12,13]^ The dominant-hand handgrip dynamometry scores (assessed 10 times) was 4 kg (interquartile range 3.3–4.8). The functional independence measure (FIM), an 18-item tool assessing physical, psychological, and social function to estimate the level of disability of a patient as well as change in patient status in response to rehabilitation or medical intervention, was 42/126. The 6-minute walking test (6MWT), used as a performance-based measure of functional exercise capacity, was not administrable, as well as the Tinetti assessment tool (that rates the degree of dependence in gait and balance) for both balance and gait components. Blood chemistry was normal, with the exception of hyperglycemia, hypoalbuminemia, and low total protein. An electromyography revealed a pattern of severe sensory-motor axonal polyneuropathy (reduced amplitude of compound muscle action potentials and sensory nerve action potentials, mildly reduced nerve conduction velocity, denervation signs, neurogenic motor unit potentials on needle electromyography). Therefore, a critical illness polyneuropathy form of ICUAW was diagnosed according to the Stevens’ criteria, that is, an axonal, sensory-motor polyneuropathy (with reduced nerve excitability and loss of axons with preserved myelin sheet, reduced amplitude of compound muscle action potentials and sensory nerve action potentials with normal or mildly reduced nerve conduction velocity on electroneurography) in the presence of a clinically detected, diffuse, symmetric weakness involving all extremities and respiratory muscles arising after the onset of critical illness (with an MRC sum score of less than 48/60 or a mean MRC score of 4 in all testable muscle groups and a dominant-hand handgrip dynamometry scores of less than 11 kg [interquartile range 10–40]).^[14,15]^

The patient was provided with a physiotherapist-assisted intensive neuro-rehabilitation program consisting of conventional and occupational therapy and respiratory physiotherapy (including passive and active range of motion, active side to side turning, cycling in bed, exercises in bed, sitting on the edge of the bed, transferring from bed to a chair, ambulation, hoist therapy, tilt table, active resistance exercises, and electrical muscle stimulation) for 180 minutes a day, 6 days a week. Furthermore, the patient was provided with high protein diet and a multi-nutritional oral program with particular protein supplement was performed.

After 2-month training (March–April 2018), the patient complained of a MRC score of 24/60, FIM of 56/126, 6MWT of 6 m, and Tinetti scale of 8/28. Because of such a still partial improvement, he was provided with a physiotherapist-supervised robotic rehabilitation protocol, in addition to the already practiced conventional and respiratory physiotherapy. Our institutional review board approved the study, and the patient provided his written informed consent to study participation and data publication.

The robotized training consisted of daily training sessions made of:

(1) gait training by using Lokomat@Nanos (Hocoma AG; Volketswil, Switzerland); and
(2) upper limb training by using Armeo^©^Spring (Hocoma AG).

All these adjunctive treatments were carried for once a day, 6 days a week, for 2 months.

The locomotor training was delivered by using a stationary system robotic exoskeleton equipped with body weight supported treadmill training and driven gait orthoses (ie, the Lokomat). The patient had first to become familiar with the device, so 3 trainings sessions were carried out also to optimize the setup. Along the sessions, the body weight support and the guidance device force were progressively reduced to the least tolerated amount starting from 100%, and the walking speed was progressively increased up to 1.2 m/s starting from 0.5 m/s. Each session consisted of a 5-minute warm-up period, a 45-minute training period (adapting body weight support, device guidance force, and walking speed every 5 minutes), and a 5-minute cool down period.

Upper limb training was delivered by using the Armeo^©^Spring, a 5 degree-of-freedom (3 in the shoulder, 1 in the elbow, and 1 in the forearm) orthosis without robotic actuators (ie, a gravity-supporting exoskeleton apparatus). This device is suitable for patients with a residual upper limb function to achieve a large active range of motion within a 3-dimensional workspace by using variable levels of gravity support guaranteed by spring mechanisms.^[16]^ The device is also equipped with a pressure-sensitive handgrip that allows the execution of graded grasp and release exercises. The patient was engaged in task-oriented motor exercises (including gross motor movement when cleaning a stove top, more precise movement when watering flowers, subtle strength-dosed movement when picking up an egg, and therapy game, for example, car racing or card playing) that were simulated in a virtual learning environment on a computer screen, also with auditory and visual performance feedback during and after practice. The patient had first to become familiar with the device, so 3 trainings sessions were carried out also to optimize the setup. Along the sessions, weight compensation, maximal active workspace, and level of exercise difficulty were adapted to the subject's ability to maintain the affected arm in a standardized position of 45° shoulder flexion and 90° elbow flexion. Each session consisted of a 5-minute warm-up period, a 45-minute training period (15 different tasks, 3 minutes per task, adapting weight compensation, maximal active workspace, and level of exercise difficulty every 5 minutes), and a 5-minute cool down period.

The patient reported no adverse events during the training. After 2 months (May–Jun 2018), he further improved in MRC (35/60), FIM (71/126), 6MWT (15m), and Tinetti scale (13/28). He was thus provided also with daily sessions of cognitive and sensory-motor training by means of the virtual reality rehabilitation system (Khymeia Group, Noventa Padovana, Italy) for other 2 months (July–August 2018). The virtual reality rehabilitation system-Evo workstation is equipped with a 3D motion-tracking system (Polhemus Liberty; Colchester, VT), a high-resolution LCD projector displaying the virtual scenarios on a large wall screen and a stabilometric and a proprioceptive/dynamic platform. The patient was sitting or standing in front of the device and interacted with the platform actively during specific, virtual sensory-motor tasks aimed at stimulating muscle strength, joint range of movement, posture stability, balance reactions, and pelvis movements. The patient had first to become familiar with the device, so 3 trainings sessions were carried out also to optimize the setup. Along the sessions, exercise difficulty was adapted in relation to the time of execution and the category of activity. Each session consisted of different types of motor tasks, including manipulating object while interacting with a virtual scenario, static balance training, supine mobilization, assisted and active exercises for trunk control and balance in a sitting position, exercises for weight shift and stepping in static and dynamic equilibrium using a stabilometric and a proprioceptive/dynamic platform.^[17,18]^

At the discharge (early September 2018, 6 months after the admission), the patient showed clear improvement in balance and ambulation (with double support) (6MWT 47 m, and Tinetti scale 22/28), muscle strength (MRC 42/60), and independency (FIM 84/126). Last, we documented a complete respiratory recovery so that oxygen therapy was withdrawn. At a 6-month follow-up visit (February 2019), the patient's clinical condition were stable.

## 3 Discussion

In-ICU rehabilitation is consolidated for maximizing functional recovery and preventing permanent limitations in patients who develop ICUAW.^[19–21]^ Noteworthy, a post-ICU intensive management of patients with ICUAW in in-patient regimen is also mandatory to improve functional outcomes, as our case suggests. To the best of our knowledge, this is the first report on robotic-aided, intensive, inpatient rehabilitative approach in a patient with ICUAW.

Our patient showed a significant improvement in gait, balance, and muscle strength yet after a 2-month intensive, conventional rehabilitation. This data agrees with those available in the literature showing that an intensive, repetitive, and early as possible mobilization reduces the negative effects of ICUAW.^[19–21]^ However, such improvements were not sufficient in our patient to completely limit the disability burden and to make the patient self-sufficient in overall mobility and the activities of daily living. According to literature, post-ICU rehabilitation is challenging, since the factors biasing functional outcome achievement are multiple (including muscle metabolism, nutrition, specific abnormalities of electrophysiological tests of peripheral nerves and muscles, muscle morphologic variations, and changes in corticospinal excitability secondary to motor unit damage), and the long-term efficacy remains uncertain (including mortality, patient functional status, quality of life, ICU or hospital LOS, duration of mechanical ventilation, or discharge disposition).^[22,23]^ Furthermore, the availability of the post-ICU rehabilitation facilities may be limited. Therefore, the post-acute rehabilitation in these patients still deserves confirmation.^[24–28]^

The rationale of adopting robotic and virtual-reality based devices in such patients stems from the fact that electromedical and substitutional devices provide patients with an intensive, assisted, repetitive, and task-oriented rehabilitation, which is essential to achieve functional recovery even when the motor deficit is extremely severe.^[11,29,30,31]^ Moreover, implementing virtual-reality based conventional and robot-aided rehabilitation allows providing the patient with a greater amount of sensorimotor information and carrying out complex and ecological tasks in a safe and fully controllable environment, as if the patient was in the real world setting.^[32,33]^ Therefore, robot-assisted and virtual reality-based rehabilitation may significantly add to conventional treatment when the latter is not sufficient to succeed functional outcomes.^[11,34–38]^ In this regard, our case suggests that combining conventional and robot-aided rehabilitation further enhances functional recovery. In fact, the patient achieved an improvement following the combined approach that was clearly larger than those obtained following conventional rehabilitation alone, and the after effects lasted up to 6 months. Nonethess, one could argue that it is not verifiable if the combined approach (traditional and robot-assisted rehabilitation) was indeed effective since the patient was previously on only conventional therapy for 2 months. Therefore, we are not able to completely rule out that the patient's improvement may result from a summation effect between the previous conventional rehabilitation alone and the next combined approach (as it was not clearly possible to provide the patient with a washout period between the approaches). Furthermore, it has been reported that patients receiving high-dose rehabilitation improve in the physical and cognitive domains of the quality of life more than those receiving low-dose rehabilitation.^[22]^ Nonetheless, robotics may have offered some advantage compared to the stand-alone conventional rehabilitation. Indeed, the magnitude of improvement following the combined approach was superior to the stand-alone conventional rehabilitation outcomes reported in the literature data,^[19–28]^ so that a summation effect seems unlikely. Instead, robotics seems to have amplified the effects of rehab training. Actually, the entire approach provided the patient with a functional improvement for at least 6 months. This may suggest that the functional improvement was not related to a simple summation effect, whose effects should be short lasting, but rather to a true recovery or compensation phenomenon based on the neuroplasticity principles of motor learning.^[29,30]^ This likely depends on the fact that robot-aided rehabilitation in such a kind of patients allows for enhancing the effects of functional training by providing the patient with highly intensive, repetitive, precise, and task-oriented motor and cognitive tasks, thus bypassing limb persistent weakness (as in our patient) by using the orthotic devices.^[11,34–38]^ In addition, also virtual reality-based devices helped to reduce motor dysfunction by emphasizing retraining and substitution of intact abilities and compensatory approaches. The greater response to the combined treatment as compared to the standalone conventional treatment may also depend on the suitability of our patient to robot-aided rehabilitation. In fact, the degree of functional impairment at the admission, patient's age, and treatment intensity are positively associated with outcomes when practicing robot-aided rehabilitation.^[39]^ However, neurorobotics has also some limitation, including general applicability to patients, patient's compliance to the orthoses, level of participation to active and assisted training, and the level of cognitive functioning (as robotic devices usually employ virtual reality feedbacks).^[11,34–38]^ Therefore, in keeping with the nature of the case-reports and the pros and cons of the interventions described above, the effectiveness and the generalizability of such combined rehabilitative approaches will require of course confirmation from ad hoc randomized clinical trials.

Using the necessary precautions for case-reports, we may hypothesize how the conventional and robotic assisted rehabilitation may be designed in patients with ICUAW in a post-ICU setting. Rehabilitation has to pursue motor deficit rehabilitation with the inspiration of motor learning principles (guaranteed by the specificity, repeatability, and task-orientation of motor practice during the training, including stretching and strengthening exercises, balance, endurance training, and enhancement of joint biomechanics), even in neuropathic patients.^[40–42]^ Such an approach aims to relieve the impairing effects of neuropathy, to improve general mobility (impairment-oriented exercises), and to enhance skilled and smooth control of movements (task-oriented exercises). In this regard, robot-aided and virtual reality-based rehabilitation play a key role. The patients who show limitations in mobility, movements, balance and stability during walking, muscle strength are ideal candidate to robot-aided and virtual reality-based rehabilitation. However, the suitability of a patient to robot-aided and virtual reality-based rehabilitation rely on many issues, including the degree of functional impairment at the admission, age, disease duration, general physical and mental status, and treatment intensity tolerance.^[39]^ Therefore, a careful patient selection is still challenging and deserves further studies specifically focusing on ICAAW rehabilitation.

In conclusion, our case suggests that patients with ICUAW could be treated intensively in in-patient regimen with robot-aided and virtual reality-based rehabilitation. Even though our approach deserves confirmation, the combined rehabilitation strategy may offer some advantage in maximizing functional recovery and containing disability, especially in those patients who complain of profound muscle weakness, are unable to stand and move upper limbs, being thus at high risk of long-term functional impairment.

## Author contributions

**Conceptualization:** Antonino Chillura.

**Data curation:** Antonino Chillura, Francesco Tartamella.

**Formal analysis:** Francesco Tartamella.

**Investigation:** Maria Francesca Pisano, Elvira Clemente, Marzia Lo Scrudato, Giuseppe Cacciato.

**Methodology:** Maria Francesca Pisano, Elvira Clemente, Marzia Lo Scrudato, Giuseppe Cacciato, Alessia Bramanti.

**Project administration:** Simona Portaro.

**Resources:** Simona Portaro.

**Software:** Simona Portaro.

**Supervision:** Rocco Salvatore Calabrò, Antonino Naro.

**Validation:** Rocco Salvatore Calabrò, Antonino Naro, Alessia Bramanti.

**Visualization:** Rocco Salvatore Calabrò.

**Writing – original draft:** Antonino Chillura, Francesco Tartamella.

**Writing – review & editing:** Antonino Naro.
